# Trends in hospital antibacterial consumption: a retrospective analysis of reimbursement data, Belgium 2017 to 2022

**DOI:** 10.2807/1560-7917.ES.2025.30.35.2500088

**Published:** 2025-09-04

**Authors:** Laura Bonacini, Julie Domen, Paul De Munter, Maya Hites, Diana Huis in‘t Veld, Antonelle Pardo, Johan Van Laethem, Dirk Vogelaers, Boudewijn Catry, Lucy Catteau

**Affiliations:** 1 Department of Epidemiology and Public Health, Sciensano, Brussels, Belgium; 2 Department of Microbiology, Immunology and Transplantation, KU Leuven, Leuven, Belgium; 3 Department of General Internal Medicine, University Hospitals Leuven, Leuven, Belgium; 4 Clinic of Infectious Diseases, Hôpital Universitaire de Bruxelles, Université Libre de Bruxelles, Brussels, Belgium; 5 Department of Internal Medicine and Infectious Diseases, Ghent University Hospital, Ghent, Belgium; 6 Laboratory of Pharmaceutical Analysis, Faculty of Medicine and Pharmacy, Research Institute for Health Sciences and Technology, University of Mons, Mons, Belgium; 7 Department of Pharmacy, CHR Haute Senne, Soignies, Belgium; 8 Internal Medicine Research Group, Vrije Universiteit Brussel (VUB), UZ Brussel, Department of Internal Medicine and Infectious Diseases, Brussels, Belgium; 9 Ghent University/Ghent University Hospital, Ghent, Belgium; 10 Department of General Internal Medicine and Infectious Diseases, AZ Delta, Roeselare, Belgium; 11 Faculty of Medicine, Université libre de Bruxelles (ULB), Brussels, Belgium; 12 Faculty of Medicine and Pharmacy, Université de Mons (UMons), Mons, Belgium; * These authors contributed equally to the work and share first authorship

**Keywords:** COVID-19, surveillance, antibiotics, hospitals

## Abstract

**BACKGROUND:**

The COVID-19 pandemic has challenged efforts to optimise rational antibacterial use due to uncertainties in treatment protocols.

**AIM:**

We investigated the impact of COVID-19 on hospital antibacterial consumption in Belgium from 2017 to 2022, relative to the general and hospitalised population.

**METHODS:**

We analysed national reimbursement data using defined daily doses (DDD) and three metrics: DDD/1,000 inhabitants/day (DID), DDD/1,000 patient days (PD) and DDD/1,000 admissions. We performed linear regressions to analyse 6-year trends (2017–2022) and estimated predicted consumption from 2020 to 2022 using the compound annual growth rate from 2017 to 2019. To assess the impact of COVID-19, we compared observed and predicted relative changes in antibacterial consumption between 2019 (pre-pandemic) and 2020 (early pandemic) and between 2019 and 2022 (late pandemic).

**RESULTS:**

From 2019 to 2020, hospital antibacterial consumption (anatomical therapeutic chemical (ATC) J01) decreased by 12% in DID but increased by 5% and 7% in DDD/1,000 PD and DDD/1,000 admissions, respectively. From 2017 to 2022, systemic antibacterials consumption declined significantly only when expressed in DID. Although all systemic antibacterial subclasses were used less than predicted between 2020 and 2022 when expressed in DID, hospital-based metrics showed higher consumption, except for macrolides and amphenicols. Broad-spectrum antibacterial consumption decreased from 2017 to 2022 when expressed in DID but fluctuated with hospital metrics, peaking in 2020, and exceeded forecasts.

**CONCLUSION:**

COVID-19 altered trends in hospital antibacterial consumption, with contrasting patterns depending on the metric used, underline the importance of hospital-specific surveillance to support targeted stewardship and preparedness efforts.

**Key public health message**

**What did you want to address in this study and why?**

We wanted to understand how antibiotic use in Belgian hospitals changed during and after the COVID-19 pandemic which significantly disrupted hospital care. We analysed antibiotic consumption using three metrics: per inhabitants, per hospital admission and per hospital day, to account for changes in patient numbers and length of stay. We also compared use in 2021 and 2022 to expected pre-pandemic levels to identify lasting effects on antibiotic use.

**What have we learnt from this study?**

Antibiotic use in hospitals appeared to decrease when measured relative to the population. However, when focusing on hospitalised patients, use increased, peaking during the COVID-19 pandemic. During the pandemic and into 2022, antibiotic use per inhabitant remained below expectations, while use per admission and per hospital day exceeded forecasts in 2020 and 2021 before returning to predicted levels in 2022.

**What are the implications of your findings for public health?**

To better understand and respond to change in antibiotic use, it is important to report both population-wide and hospital-specific data. Our findings show that while a pandemic can cause temporary change in hospital antibiotic use, consumption to return to previous trends within 2022. To prevent overuse and ensure quicker return to safe prescribing, stewardship efforts should be reinforced during and after health crises.

## Introduction

Antimicrobial resistance (AMR) remains a critical global health problem, exacerbated by excessive and inappropriate use of antibiotics. Murray et al. developed an approach to estimate the burden of AMR, reporting that in 2019, 4.95 million deaths were associated with AMR worldwide [1]. In July 2022, the European Commission and European Union (EU) countries identified AMR as one of the top three priority health threats, prompting coordinated efforts to promote responsible and prudent antimicrobial use [2].

Since 2001, Belgium has been participating in the European Surveillance of Antimicrobial Consumption Network (ESAC-Net) coordinated by the European Centre for Disease Prevention and Control (ECDC). This network provides a standardised methodology for comprehensive monitoring of antimicrobial consumption (AMC) across Europe, encompassing both community and hospital sectors, using defined daily doses (DDD) per 1,000 inhabitants per day (DID) as a common metric.

In parallel, Belgium launched its first national hospital antimicrobial consumption surveillance system in 2007, enhancing data granularity and enabling benchmarking efforts [3]. Subsequently, the Belgian Hospitals – Surveillance of Antimicrobial Consumption (BeH-SAC) was introduced in 2018 with enhanced methodologies, integrating reimbursement data from the National Institute for Health and Disability Insurance (NIHDI) and implementing a new online reporting system (Healthstat.be). These improvements ensured comprehensive and streamlined data collection by using existing validated data, thereby reducing human workload [3].

Aligned with European directives, Belgium emphasised the importance of optimised antimicrobial consumption surveillance in a national action plan to prevent AMR [4]. For the Belgian hospital sector, strategic objectives for the period 2020 to 2024 included: (i) a gradual decrease in total AMC, to be monitored nationally with comparison to antimicrobial consumption in other countries, and (ii) a reduction in broad-spectrum antibacterial consumption as a proportion of total AMC along with a 5% increase in the use of narrow-spectrum antibacterials, as classified by the World Health Organization (WHO) Access, Watch, Reserve (AWaRE) system [4,5].

A common way to measure antibacterial consumption is to divide the total volume of antibacterials used by the total number of inhabitants in a country, a method known as using a population-based denominator. While this approach is useful for assessing national trends, it may not accurately reflect antimicrobial use in hospitals. This was highlighted during the COVID-19 pandemic in 2020 which profoundly impacted hospital settings, leading to a surge in infection-related admissions and an initial widespread use of empirical antibiotics such as azithromycin [6-8]. Fluctuations in hospital length of stay (LOS) and changes in patient demographics further emphasised the limitations of this metric [8,9]. As a result, hospital-specific denominators, such as the number of hospital admissions or the total number of days patients spend in the hospital (i.e. patient days), were recommended as more precise measures for tracking antibacterial consumption in hospitalised patients [10,11].

This study aimed to evaluate the impact of the COVID-19 pandemic on antibacterial consumption in Belgian hospitals between 2020 and 2022. To do so, we assessed AMC in hospitals as a proportion of the total population and as a proportion of hospitalised patients. Where AMC per 1,000 inhabitants per day reflects changes in antibiotic consumption in the overall population, AMC per 1,000 patient days represents the amount of antibiotics consumed per day of hospitalisation, while AMC per 1,000 admissions reflects the amount of antibiotics used per hospital stay. We compared actual consumption with forecasted estimates based on pre-pandemic data. The analysis is structured in three parts: (i) the early impact of the pandemic, where we examine deviations in AMC between 2019 and 2020 and compare it with forecasted consumption, (ii) the late impact of the pandemic, where we focus this comparison on the change observed between 2019 and 2022, and (iii) a comparison between observed and predicted trends between 2017 and 2022.

## Methods

### Data collection and metrics

We obtained data on antibacterial consumption in Belgian hospitals from the National Institute for Health and Disability Insurance (NIHDI) and processed the data as part of the ESAC-Net and BeH-SAC projects [12,13]. The dataset, covering the period from 2017 to 2022, includes only reimbursed antimicrobial agents. We applied an extrapolation from 99% to 100%, given that approximately 99% of the Belgian population has health insurance [14]. The ESAC-Net dataset encompasses all Belgian hospitals, including chronic, psychiatric and acute care hospitals (n = 170), while the BeH-SAC dataset specifically focuses on all acute care hospitals (n = 103) [3,15]. In Belgium, both public and private hospitals receive the same government funding for medication reimbursement. In 2019, 28% of the 103 hospitals were public, managed by government entities, while 72% were private, non-profit associations with historical ties to religious or mutual insurance organisations. Specialised private clinics, lacking hospital pharmacies, are excluded from hospital surveillance and their antimicrobial prescriptions are captured in national ambulatory AMC surveillance [16]. 

Antibacterials were categorised using the Anatomical Therapeutic Chemical (ATC) classification system. We employed the defined daily dose (DDD) methodology, developed by the World Health Organization Collaborating Centre for Drug Statistics and Methodology (Oslo, Norway), to standardise the quantification of antibacterial consumption using the 2024 ATC Index [17].

The population data for Belgium were obtained from Eurostat, which displays annual data on the population as on 1 January each year [18]. Yearly data on the number of patient days (counting both the day of admission and discharge as separate days) and the number of admissions were obtained from the Research, Development and Quality department of the NIHDI. These data represent all invoiced stays in Belgian acute care hospitals from 2017 to 2022 and were collected per hospital, based on a unique NIHDI number. We determined the yearly length of stay by dividing the number of patient days by the number of admissions, aggregated at hospital level. We calculated the median length of stay in Belgium from 2017 to 2022 as the median across all acute care hospitals (n = 103).

Three metrics were used to calculate hospital antibacterial consumption rates: (i) DDD per 1,000 inhabitants per day (DID), using reimbursed DDD data from all hospitals (ESAC-Net, n = 170) extrapolated to cover the entire Belgian population using Eurostat population data; (ii) DDD per 1,000 patient days (PD) and (iii) per 1,000 admissions, metrics specific to acute care hospitals (BeH-SAC, n = 103) using reimbursed DDD and hospital data from NIHDI, excluding psychiatric and 1-day hospitalisation wards. Since 2019, detailed patient day data for chronic care hospitals have no longer been collected. By consequence, antimicrobial consumption metrics calculated per 1,000 admissions and per 1,000 patient days, which more accurately reflect actual care activity, can only be applied to acute care hospitals.

### Data analysis

We used linear regressions to examine trends in denominators (inhabitants, patient days, admissions), and median length of stay across all acute care hospitals from 2017 to 2022.

The compound annual growth rate (CAGR) from the period before the COVID-19 pandemic (2017–2019) was used to forecast antibacterial consumption for the years 2020 to 2022. The CAGR represents the mean annual percentage change in consumption over the specified period, assuming a constant rate of growth or decline. It is calculated using the formula:

CAGR = (EV/SV)^(1/n) – 1

where EV is the ending value, SV is the starting value and n is the number of years or periods [19].

Relative changes in consumption were calculated to assess the immediate impact of COVID-19, comparing 2019 (pre-pandemic) with 2020 (pandemic onset) and the possible long-term effect of COVID-19 by comparing 2019 with 2022 (late pandemic). In addition, deviations between actual and predicted consumption for 2020 and 2022 were calculated and expressed as relative percentages to quantify these differences.

Trends in actual and forecasted consumption (DID, DDD/1,000 PD and DDD/1,000 admissions) were analysed using linear regressions from 2017 to 2022 of all antibacterials at ATC 2 (J01: Antibacterials for systemic use) and ATC 3 levels (pharmacological subclasses). We used ATC 5 level to analyse consumption of azithromycin (J01FA10), and ESAC-Net defined-broad-spectrum antibacterials (piperacillin with enzyme inhibitor (J01CR05), third-generation cephalosporins (J01DD), fourth-generation cephalosporins (J01DE), monobactams (J01DF), carbapenems (J01DH), fluoroquinolones (J01MA), glycopeptides (J01XA), polymyxins (J01XB), and linezolid (J01XX08). Daptomycin (J01XX09) and tedizolid (XX11) are not available in Belgium and were therefore not included [12,20].

We conducted a sensitivity analysis in which we calculated the DID for acute care hospitals included in the BeH-SAC dataset and performed a regression analysis for J01 to address concerns regarding the comparability of hospital groups between the ESAC-Net dataset (used for DID) and the BeH-SAC dataset (used for DDD/1,000 PD and DDD/1,000 admissions).

Statistical significance was noted for p values < 0.05. Analyses were conducted using SAS Enterprise Guide software version 7.13 (SAS Institute Inc., Cary, United States).

## Results

### Impact of COVID-19 on denominators

From 2017 to 2022, the Belgian population increased steadily (Table 1). In contrast, the two hospital metrics, admissions and patient days, declined sharply in 2020, followed by a gradual recovery in 2021 and 2022. Notably, while the number of admissions had returned to pre-pandemic levels by 2022, patient days had not. Meanwhile, the median length of stay decreased significantly over the period, with the most pronounced reductions in 2021 and 2022.

**Table 1:** Trends in population and hospital activity metrics, Belgium, 2017–2022

Denominator	2017	2018	2019	2020	2021	2022	p value	Trend
Inhabitants (× 10^5^)	114	114	115	115	116	116	**< 0.01**	↑
Admissions (× 10^5^)	17.5	17.6	17.7	14.8	15.9	17.6	0.58	NS
Patient days (× 10^5^)^a^	118	118	117	100	103	107	0.07	NS
Length of stay (days)^b^	6.58	6.53	6.43	6.54	6.17	5.83	**0.03**	↓

NS: not significant; ↓: significant decrease ; ↑: significant increase

^a^ Excluding psychiatric and 1-day hospitalisation wards.

^b^ Median of yearly length of stay calculated by dividing the number of patient days by the number of admissions, aggregated at hospital level. 6-year trend analyses were performed using linear regressions.

p values < 0.05 (in bold) were considered as a significant trend.

### Early pandemic antibacterial consumption (2020 vs 2019)

**Figure 1:**
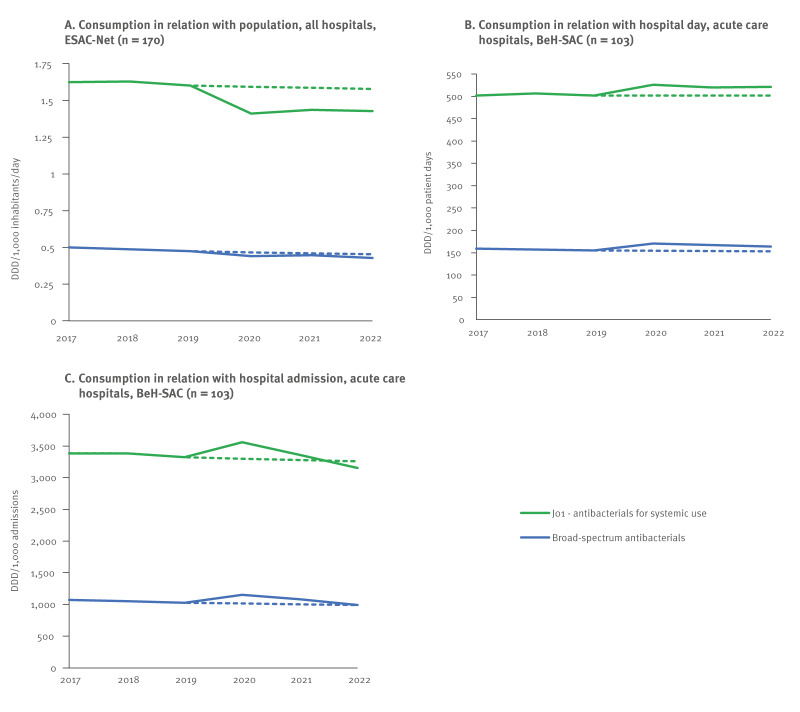
Actual and forecasted hospital consumption trends of systemic (J01) and broad-spectrum antibacterials expressed in DID (n = 170), DDD per 1,000 PD and DDD per 1,000 admissions (n = 103), Belgium, 2017–2022

DDD: defined daily doses; DID: defined daily doses per 1,000 inhabitants; PD: patient days.

**Figure 2:**
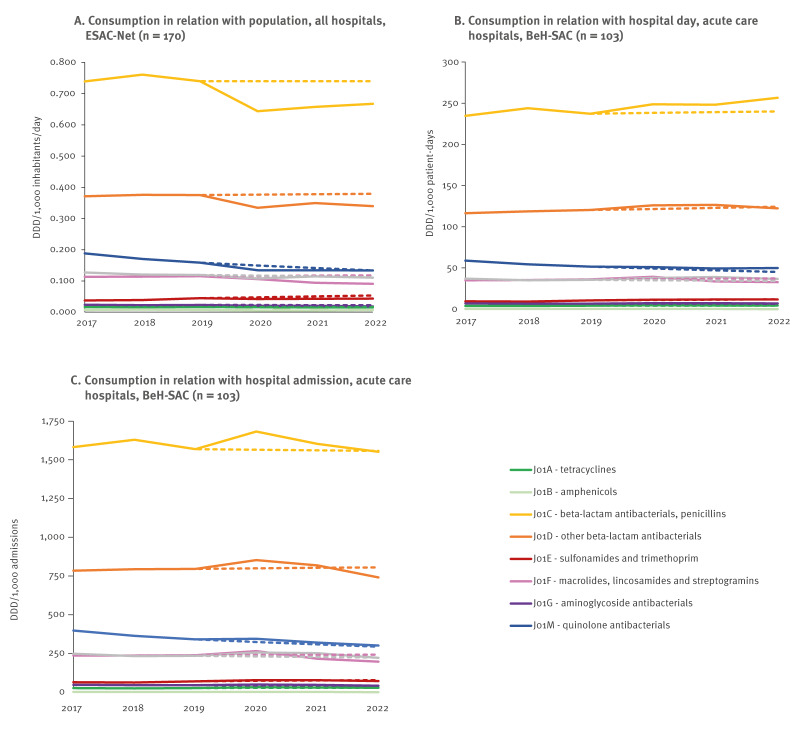
Actual and forecasted hospital consumption trends of systemic antibacterial subclasses (ATC-3 level) expressed in DID (n = 170), DDD per 1,000 PD and DDD per 1,000 admissions (n = 103), Belgium, 2017–2022

DDD: defined daily doses; DID: defined daily doses per 1,000 inhabitants; PD: patient days.

While the overall forecasts anticipated only minimal changes between 2019 and 2020, actual hospital systemic antibacterial consumption (J01) decreased by 11.8% in DID (Table 2). In contrast, consumption increased by 4.7% in DDD per 1,000 PD, and by 7.2% in DDD per 1,000 admissions (Figure 1). The same was observed for all ATC-3 level subclasses: a decline in DID, but an increase in hospital-based metrics, except for the consumption of quinolone antibacterials (J01M) which also decreased in DDD per 1,000 PD (Table 2, Figure 2).

**Table 2:** Actual and forecasted hospital consumption of systemic antibacterials (ATC J01) expressed in DID (n = 170), DDD per 1,000 PD and DDD per 1,000 admissions (n = 103), Belgium, 2017–2022

Metric	Actual antibacterial consumption										CAGR	Forecasted antibacterial consumption							Relative difference between forecasted and actual (%)	
	2017	2018	2019	2020	2021	2022	p value	Trend	Relative change (%)			2020	2021	2022	p value	Trend	Relative change (%)		2021	2022
									2019–2020	2019–2022							2019–2020	2019–2022		
Antibacterials for systemic use (J01)																				
DID	1.625	1.628	1.601	1.411	1.435	1.427	**0.02**	↓	−11.8	−10.9	-0.5	1.593	1.585	1.577	**0.00**	↓	−0.5	−1.5	−10.4	−10.5
DDD/1,000 PD	502.0	506.8	502.1	525.8	519.9	521.1	0.05	NS	4.7	3.8	0.0	502.1	502.1	502.1	**0.00**	↓	0.0	0.0	3.4	3.6
DDD/1,000 admissions	3385.1	3384.0	3320.8	3559.6	3357.4	3152.6	0.42	NS	7.2	−5.1	−0.6	3299.6	3278.6	3257.7	0.47	NS	−0.6	−1.9	2.3	−3.3
Tetracyclines (J01A)																				
DID	0.017	0.016	0.017	0.016	0.015	0.015	**0.04**	↓	−5.2	−6.9	−0.1	0.017	0.017	0.017	0.60	NS	−0.1	−0.3	−7.3	−7.1
DDD/1,000 PD	3.9	3.7	4.0	4.7	4.5	4.5	0.06	NS	17.4	11.2	1.2	4.1	4.1	4.2	0.12	NS	1.2	3.7	7.7	6.7
DDD/1,000 admissions	26.1	24.7	26.6	31.9	28.8	27.0	0.35	NS	20.2	1.8	0.6	26.7	26.9	27.0	**0.03**	NS	0.6	1.8	6.7	−0.0
Amphenicols (J01B)																				
DID	0.007	0.008	0.008	0.006	0.006	0.007	0.14	NS	−27.7	−14.8	1.4	0.008	0.008	0.008	0.07	NS	1.4	4.4	−42.7	−22.5
DDD/1,000 PD	0.1	0.1	0.1	0.1	0.0	0.0	**0.01**	↓	20.0	−58.9	−17.2	0.1	0.0	0.0	**0.00**	↓	−17.2	−43.1	−62.2	−38.3
DDD/1,000 admissions	0.8	0.7	0.4	0.5	0.2	0.2	**0.01**	↓	22.8	−62.4	−17.7	0.4	0.3	0.2	**0.00**	↓	−17.7	−44.2	−64.0	−48.3
Beta-lactam antibacterials, penicillins (J01C)																				
DID	0.739	0.761	0.740	0.644	0.658	0.668	0.05	NS	−13.0	−9.8	0.0	0.740	0.740	0.740	0.48	NS	0.0	0.0	−12.5	−10.9
DDD/1,000 PD	234.6	243.9	237.3	248.6	248.1	256.7	**0.02**	↑	4.8	8.2	0.4	238.2	239.2	240.1	0.15	NS	0.4	1.2	3.6	6.5
DDD/1,000 admissions	1582.1	1629.0	1569.8	1683.1	1602.4	1553.0	0.81	NS	7.2	−1.1	−0.3	1565.7	1561.7	1557.6	0.65	NS	−0.3	−0.8	2.5	−0.3
Other beta-lactam antibacterials (J01D)																				
DID	0.371	0.376	0.375	0.335	0.350	0.340	0.06	NS	−10.8	−9.5	0.3	0.376	0.378	0.379	**0.01**	↑	0.3	1.1	−8.0	−11.6
DDD/1,000 PD	116.4	118.7	120.3	126.1	126.6	122.3	0.07	NS	4.8	1.7	1.1	121.6	122.9	124.2	**0.00**	↑	1.1	3.3	2.9	−1.6
DDD/1,000 admissions	785.1	792.8	795.4	853.3	817.4	739.9	0.80	NS	7.3	−7.0	0.4	798.9	802.4	805.9	**0.00**	↑	0.4	1.3	1.8	−8.9
Sulfonamides and trimethoprim (J01E)																				
DID	0.037	0.039	0.045	0.042	0.043	0.044	0.09	NS	−6.5	−2.7	6.3	0.048	0.051	0.054	**0.00**	↑	6.3	20.0	−18.5	−23.3
DDD/1,000 PD	9.3	9.2	10.6	11.3	11.8	11.8	**0.00**	↑	7.3	11.7	4.2	11.0	11.4	11.9	**0.00**	↑	4.2	13.0	3.0	−1.2
DDD/1,000 admissions	63.0	61.2	69.8	76.6	76.2	71.3	0.07	NS	9.8	2.1	3.5	72.2	74.8	77.4	**0.00**	↑	3.5	10.9	1.9	−8.5
Macrolides, lincosamides and streptogramins (J01F)																				
DID	0.113	0.114	0.115	0.106	0.094	0.091	**0.01**	↓	−8.3	−21.4	0.7	0.116	0.117	0.118	**0.00**	↑	0.7	2.1	−23.9	−29.9
DDD/1,000 PD	35.0	35.2	36.1	39.2	33.4	32.6	0.52	NS	8.5	−9.6	1.0	36.5	36.8	37.2	**0.00**	↑	1.0	3.1	−10.1	−14.1
DDD/1,000 admissions	236.2	235.4	238.8	265.2	216.0	197.4	0.28	NS	11.1	−17.3	0.4	239.7	240.5	241.4	**0.00**	↑	0.4	1.1	−11.4	−22.3
Azithromycin (J01FA10)																				
DID	0.027	0.029	0.033	0.038	0.030	0.030	0.44	NS	15.8	−6.5	6.9	0.035	0.037	0.040	**0.04**	↑	6.9	22.2	−22.4	−30.6
DDD/1,000 PD	7.2	8.0	8.9	12.8	9.5	9.7	0.22	NS	43.4	9.5	7.2	9.5	10.2	11.0	**0.00**	↑	7.2	23.2	−7.6	−12.6
DDD/1,000 admissions	48.7	53.2	58.9	86.4	61.4	59.0	0.41	NS	46.7	0.1	6.5	62.7	66.8	71.2	**0.01**	↑	6.5	20.8	−8.8	−20.7
Aminoglycoside antibacterials (J01G)																				
DID	0.0	0.0	0.0	0.0	0.0	0.0	**0.00**	↓	−10.7	−20.2	−1.1	0.023	0.023	0.022	**0.00**	↓	−1.1	−3.3	−11.2	−21.1
DDD/1,000 PD	7.0	6.7	6.7	7.2	7.1	6.8	0.77	NS	8.0	0.6	−1.3	6.6	6.5	6.5	**0.00**	↓	−1.3	−3.8	7.9	4.4
DDD/1,000 admissions	47.1	44.6	44.4	49.1	45.9	40.8	0.39	NS	10.5	−8.0	−1.9	43.5	42.7	41.9	**0.00**	↓	−1.9	−5.7	6.9	−2.5
Quinolone antibacterials (J01M)																				
DID	0.189	0.171	0.159	0.134	0.135	0.134	**0.01**	↓	−15.5	−15.6	−5.6	0.150	0.141	0.133	**0.00**	↓	−5.6	−15.9	−5.0	0.4
DDD/1,000 PD	58.8	54.3	51.5	51.0	49.3	49.8	**0.01**	↓	−1.1	−3.3	−4.3	49.3	47.2	45.1	**0.01**	↓	−4.3	−12.4	4.4	9.4
DDD/1,000 admissions	396.6	362.4	340.7	345.0	318.7	301.3	**0.00**	↓	1.2	−11.6	−4.9	323.9	307.9	292.7	**0.04**	↓	−4.9	−14.1	3.4	2.8
Other antibacterials (J01X)																				
DID	0.127	0.121	0.120	0.108	0.115	0.111	**0.03**	↓	−9.3	−7.4	−2.1	0.117	0.115	0.112	**0.00**	↓	−2.1	−6.1	−0.0	−1.3
DDD/1,000 PD	36.8	34.9	35.5	37.6	39.0	36.7	0.32	NS	6.0	3.2	−1.2	35.1	34.7	34.2	**0.00**	↓	−1.2	−3.5	11.1	6.6
DDD/1,000 admissions	248.2	233.2	234.8	254.9	251.9	221.7	0.65	NS	8.5	−5.6	−1.8	230.5	226.3	222.2	**0.47**	↓	−1.8	−5.4	10.1	−0.2

CAGR: compound annual growth rate; DDD: defined daily doses; DID: defined daily doses per 1,000 inhabitants; PD: patient days; **NS:** not significant; ↓: significant decrease; ↑: significant increase.

DID were calculated using reimbursed DDD from all Belgian hospitals (ESAC-Net, n = 170) and population data from Eurostat. DDD/1,000 PD and DDD/1,000 admissions, were calculated using reimbursed DDD from all acute care hospitals (BeH-SAC, n = 103) excluding psychiatric and 1-day hospitalisation wards. Corresponding hospital denominators based on invoiced stays were obtained from the National Institute for Health and disability Insurance. A PD corresponds to an overnight stay, including hospital admission and discharge days [31]. The CAGR was calculated using data from 2017 to 2019 data to forecast antibacterial consumption for the years 2020 to 2022. The 6-year trend analyses were performed on both the actual consumption and forecasted consumption using linear regressions. p values < 0.05 (in bold) were considered as a significant trend. Figures in this table have been rounded for presentation. However, all statistical analyses and significance testing were conducted using unrounded data.

Solid lines: actual antibacterial consumption; dotted lines: predicted antibacterial consumption based on the compound annual growth rate calculated from 2017 to 2019 data.

Solid lines: actual antibacterial consumption; dotted lines: predicted antibacterial consumption based on compound annual growth rate calculated from 2017 to 2019 data.

Consumption of amphenicols (J01B), penicillins (J01C), other β-lactam antibacterials (J01D), sulfonamides and trimethoprim (J01E), and macrolides, lincosamides, streptogramins (J01F) dropped between 2019 and 2020 using DID, which contrasted the forecasted increases (Table 2). While decreases were forecasted for amphenicols (J01B), aminoglycosides (J01G) and other antibacterials (J01X) using hospital-based metrics, actual increases were recorded. Consumption of azithromycin (J01FA10) increased more than forecasted in all three metrics (Table 2).

**Figure 3:**
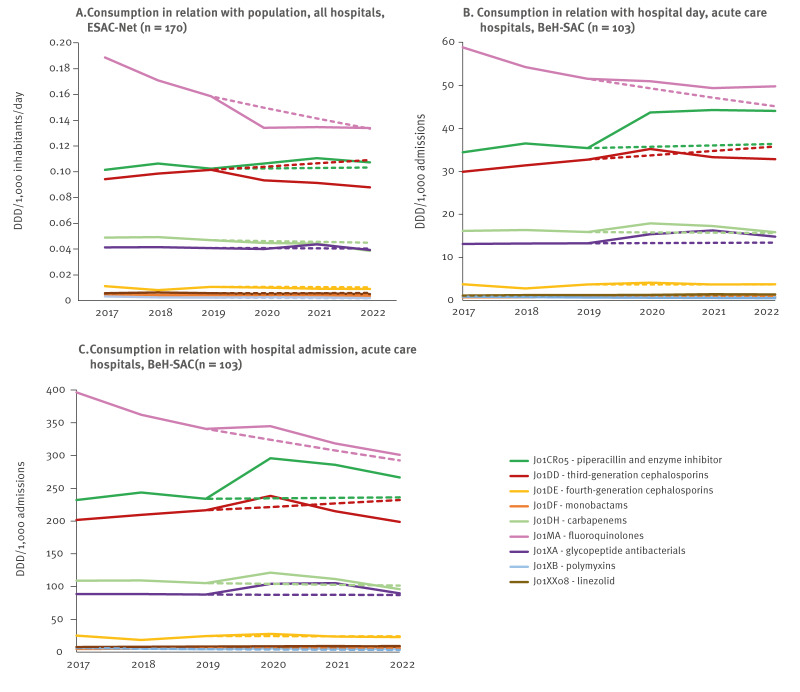
Actual and forecasted hospital consumption trends of broad-spectrum antibacterials subclasses (ATC-4 or ATC-5 level) expressed in DID (n = 170), DDD per 1,000 PD and DDD per 1,000 admissions (n = 103), Belgium, 2017–2022

DDD: defined daily doses; DID: defined daily doses per 1,000 inhabitants; PD: patient days.

Broad-spectrum antibacterial consumption also decreased in DID and increased using hospital-based metrics, deviating from the stable consumption forecasted across all three metrics (Table 2, Figure 1). Only piperacillin and enzyme inhibitor (J01CR05) and polymyxins (J01XB) were consumed more in 2020 compared with 2019 when using DID. Polymyxins (J01XB) were even forecasted to decrease but instead showed a significant rise. Consumption of third-generation cephalosporines (J01DD) decreased in contrast to forecasted increases (Table 2) (Table 2, Figure 3).

Solid lines: actual antibacterial consumption; dotted lines: predicted antibacterial consumption based on compound annual growth rate calculated from 2017 to 2019 data.

### Late-pandemic antibacterial consumption (2022 vs 2019)

In 2022, β-lactams constituted the majority of hospital antibacterial consumption, with penicillins (J01C) accounting for 49.2% of total consumption, other β-lactam antibacterials (J01D) for 23.4%, and quinolones (J01M) for 9.6%. Broad-spectrum antibacterials represented 31.5% of total consumption, with fluoroquinolones (J01MA, 9.6%) and piperacillin with enzyme inhibitor (J01CR05, 8.4%) most commonly used.

Between 2019 and 2022, hospital systemic antibacterial consumption (J01) in DID decreased by 10.9%, exceeding the forecasted 1.5% reduction. Hospital-based metrics showed mixed patterns: DDD per 1,000 PD increased by 3.8%, while no change was forecasted, and DDD per 1,000 admissions decreased by 5.1%, a sharper decline than the forecasted 1.9% reduction (Table 2, Figure 1). All systemic antibacterial subclasses decreased more sharply than forecasted between 2019 and 2022 in DID (Table 2, Figure 2). Using hospital-based metrics, we mainly noted a decrease in both macrolides (J01F) and quinolones (J01M) consumption. However, quinolones (J01M) remained above forecasted consumption levels, unlike macrolides (J01F), where actual consumption was below the forecasted levels. Azithromycin (J01FA10) consumption in particular spiked in 2020 but stabilised in 2022 (Table 2). The minimal increase of 0.1% between 2019 and 2022 contrasted the predicted 20.8% increase (Table 2).

Broad-spectrum antibacterial consumption decreased more sharply in DID between 2019 and 2022 than forecasted (Figure 1). When measured as DDD per 1,000 admissions, consumption decreased slightly as forecasted, but the DDD per 1,000 PD metric increased, diverging from forecasts (Table 2, Figure 1). Consumption of piperacillin with enzyme inhibitor (J01CR05) and glycopeptides (J01XA) was still higher than in 2019 and higher than forecasted. Third-generation cephalosporins (J01DD) and monobactams (J01DF) fell below predicted consumption in both DID and hospital-based metrics in 2022. For fourth-generation cephalosporines (J01DE), carbapenems (J01DH) and linezolid (J01XX08) consumption in 2022 was also lower-than-expected, as indicated by DID and DDD per 1,000 admissions (Table 2).

Focusing on the national strategic objectives for the period 2020 to 2024, we observed a slight reduction in broad-spectrum antibacterial consumption as a proportion of total AMC, from 31.2% in 2020 to 29.9% in 2022 (ESAC-Net data). In addition, the consumption of narrow-spectrum antibacterials increased with 2%, rising from 0.75 DID in 2020 to 0.77 DID in 2022.

### Comparing actual and forecasted trends

From 2017 to 2022, a statistically significant negative trend was only detected in DID for systemic antibacterials (J01), although negative trends were forecasted using both DID and DDD per 1,000 PD (Table 2). Antibacterial consumption using DDD per 1,000 PD remained above predicted levels throughout the period, whereas consumption in DDD per 1,000 admissions consumption increased in 2020 before returning closer to predicted levels in 2021 and slightly below in 2022 (Figure 1). Quinolone antibacterials (J01M) were the only subclass with statistically significant decreasing trends across all three metrics, consistent with predictions, although the actual decline was less pronounced than predicted.

In DID, significant decreases were observed for tetracyclines (J01A), macrolides, lincosamides and streptogramins (J01F), aminoglycosides (J01G), and other antibacterials (J01X). The decrease in macrolides, lincosamides and streptogramins (J01F) contrasts with the increasing trend predicted for this subclass. Using both hospital-based metrics, significant decreasing trends were observed for quinolone antibacterials (J01M) and amphenicols (J01B), while significant increasing trends were noted for sulfonamides and trimethoprim (J01E) using DDD per 1,000 admissions, and for β-lactam antibacterials, penicillins (J01C) when measured as DDD per 1,000 PD.

Consumption of broad-spectrum antibacterials showed a significant decreasing trend in DID only, while declines were forecasted across all three metrics (Table 3). Using DDD per 1,000 PD, monobactams (J01DF) and linezolid (J01X08) showed increasing trends as predicted, while the increasing trend of piperacillin and enzyme inhibitor (J01CR05) was unpredicted (Table 3, Figure 1, Figure 3).

**Table 3:** Actual and forecasted hospital consumption of broad-spectrum antibacterials expressed in DID (n = 170), DDD per 1,000 PD, DDD per 1,000 admissions (n = 103), Belgium, 2017–2022

Metric	Actual antibacterial consumption										CAGR	Forecasted antibacterial consumption							Relative difference between predicted and actual (%)	
	2017	2018	2019	2020	2021	2022	p value	Trend	Relative change (%)			2020	2021	2022	p value	Trend	Relative change (%)		2021	2022
									2019–2020	2019–2022							2019–2020	2019–2022		
Broad-spectrum antibacterials (total)																				
DID	0.499	0.488	0.474	0.441	0.446	0.427	**0.00**	↓	−7.0	−9.9	−1.7	0.467	0.460	0.454	**0.00**	↓	−1.5	−4.2	−3.1	-6.3
DDD/1,000 PD	158.9	157.3	155.2	170.3	167.3	164.1	0.18	NS	9.7	5.7	−0.8	154.3	153.5	152.8	**0.00**	↓	−0.6	−1.5	8.3	6.8
DDD/1,000 admissions	1071.7	1050.5	1026.8	1152.5	1080.6	992.6	0.74	NS	12.2	−3.3	−1.4	1014.1	1002.4	991.6	**0.00**	↓	−1.2	−3.4	7.2	0.1
Piperacillin and enzyme inhibitor (J01CR05)																				
DID	0.101	0.106	0.102	0.106	0.111	0.107	0.11	NS	3.9	4.8	0.3	0.103	0.103	0.103	0.94	NS	0.3	0.9	6.9	3.7
DDD/1,000 PD	34.4	36.5	35.4	43.7	44.2	44.0	**0.01**	↑	23.4	24.4	0.9	35.7	36.0	36.4	0.89	NS	0.9	2.8	18.5	17.4
DDD/1,000 admissions	232.2	243.7	234.1	295.8	285.8	266.4	0.12	NS	26.3	13.8	0.3	234.7	235.4	236.0	0.20	NS	0.3	0.8	17.6	11.4
Third-generation cephalosporins (J01DD)																				
DID	0.094	0.099	0.101	0.093	0.091	0.088	0.14	NS	−7.9	−13.3	2.5	0.104	0.107	0.109	**0.00**	↑	2.5	7.6	−16.6	−24.1
DDD/1,000 PD	29.9	31.4	32.7	35.2	33.3	32.8	0.14	NS	7.7	0.4	3.0	33.7	34.7	35.8	**0.00**	↑	3.0	9.4	−4.4	−9.0
DDD/1,000 admissions	201.6	209.6	216.4	238.4	214.9	198.7	0.87	NS	10.2	−8.2	2.4	221.5	226.8	232.2	**0.00**	↑	2.4	7.3	−5.5	−16.9
Fourth-generation cephalosporins (J01DE)																				
DID	0.011	0.008	0.011	0.010	0.009	0.009	0.44	NS	−5.9	−15.9	−1.3	0.011	0.010	0.010	0.83	NS	−1.3	−4.0	−13.2	−14.2
DDD/1,000 PD	3.7	2.8	3.7	4.1	3.7	3.8	0.45	NS	10.7	1.2	−0.2	3.7	3.7	3.7	0.67	NS	−0.2	−0.6	−0.3	1.8
DDD/1,000 admissions	25.2	18.6	24.5	27.8	23.8	22.7	0.83	NS	13.3	−7.5	-0.9	24.3	24.1	23.9	0.49	NS	−0.9	−2.5	−1.4	−5.3
Monobactams (J01DF)																				
DID	0.005	0.004	0.004	0.004	0.004	0.004	**0.02**	↓	−6.2	−18.9	−0.3	0.004	0.004	0.004	**0.01**	↓	−0.3	−1.0	−6.1	−22.0
DDD/1,000 PD	0.7	0.7	0.8	0.9	1.0	0.9	**0.04**	↑	8.6	7.7	3.4	0.8	0.9	0.9	**0.00**	↑	3.4	10.7	12.8	−2.7
DDD/1,000 admissions	4.9	4.9	5.3	5.9	6.3	5.2	0.20	NS	11.1	−1.5	2.8	5.4	5.6	5.7	**0.00**	↑	2.8	8.6	11.9	−10.2
Carbapenems (J01DH)																				
DID	0.049	0.049	0.047	0.045	0.044	0.039	**0.01**	↓	−4.6	−17.7	−1.4	0.046	0.046	0.045	**0.00**	↓	−1.4	−4.0	−3.2	−16.7
DDD/1,000 PD	16.2	16.4	15.9	17.9	17.3	15.8	0.71	NS	12.6	−0.4	−0.5	15.8	15.7	15.7	**0.00**	↓	−0.5	−1.6	9.0	1.2
DDD/1,000 admissions	109.1	109.4	105.3	121.3	111.7	95.9	0.59	NS	15.2	−8.9	−1.2	104.0	102.8	101.6	**0.02**	↓	−1.2	−3.5	8.0	−5.9
Fluoroquinolones (J01MA)																				
DID	0.189	0.171	0.159	0.134	0.135	0.134	**0.01**	↓	−15.5	−15.6	−5.6	0.150	0.141	0.133	**0.00**	↓	−5.6	−15.9	-5.0	0.4
DDD/1,000 PD	58.8	54.3	51.5	51.0	49.3	49.8	**0.01**	↓	−1.1	−3.3	−4.3	49.3	47.2	45.1	**0.00**	↓	−4.3	−12.4	4.4	9.4
DDD/1,000 admissions	396.6	362.4	340.7	345.0	318.7	301.3	**0.00**	↓	1.2	−11.6	−4.9	323.9	307.9	292.7	**0.00**	↓	−4.9	−14.1	3.4	2.8
Glycopeptide antibacterials (J01XA)																				
DID	0.041	0.042	0.041	0.040	0.044	0.039	0.78	NS	−2.0	−3.5	-0.4	0.041	0.041	0.040	**0.01**	↓	−0.4	−1.2	7.3	−2.4
DDD/1,000 PD	13.1	13.3	13.3	15.4	16.3	14.8	0.06	NS	15.7	11.6	0.4	13.3	13.4	13.4	**0.00**	↓	0.4	1.2	17.8	9.3
DDD/1,000 admissions	88.5	88.5	87.8	104.0	105.2	89.6	0.35	NS	18.4	2.0	-0.2	87.6	87.4	87.2	**0.00**	↑	−0.2	−0.7	16.9	2.7
Polymyxins (J01XB)																				
DID	0.003	0.003	0.002	0.003	0.003	0.002	**0.04**	↓	9.6	−19.7	−8.7	0.002	0.002	0.002	**0.00**	↓	−8.7	−24.0	19.0	5.3
DDD/1,000 PD	0.9	0.8	0.7	0.8	0.8	0.7	0.34	NS	23.6	6.6	−9.5	0.6	0.5	0.5	**0.00**	↓	−9.5	−25.9	30.2	30.5
DDD/1,000 admissions	6.1	5.1	4.4	5.6	5.0	4.3	0.19	NS	26.5	−2.5	−10.1	4.0	3.6	3.2	**0.00**	↓	−10.1	−27.3	29.4	25.5
Linezolid (J01XX08)																				
DID	0.006	0.006	0.006	0.005	0.006	0.005	0.14	NS	-13.0	-11.7	0.5	0.006	0.006	0.006	0.86	NS	0.5	1.6	−5.1	−15.1
DDD/1,000 PD	1.1	1.2	1.3	1.3	1.4	1.4	**0.00**	↑	4.5	12.9	3.7	1.3	1.3	1.4	**0.00**	↑	3.7	11.6	5.1	1.1
DDD/1,000 admissions	7.6	8.2	8.3	8.8	9.2	8.5	0.06	NS	7.0	3.3	3.1	8.5	8.8	9.1	**0.00**	↑	3.1	9.5	4.1	-6.0

CAGR: compound annual growth rate; DDD: defined daily doses; DID: defined daily doses per 1,000 inhabitants; PD: patient days; **NS:** not significant; ↓: significant decrease; ↑: significant increase.

DID were calculated using reimbursed DDD from all Belgian hospitals (ESAC-Net, n = 170) and population data from Eurostat. DDD/1,000 PD and DDD/1,000 admissions, were calculated using reimbursed DDD from all acute care hospitals (BeH-SAC, n = 103) excluding psychiatric and 1-day hospitalisation wards and corresponding hospital data based on invoiced stays were obtained from the National Institute for Health and disability Insurance. A patient-day corresponds to an overnight stay, including hospital admission and discharge days [31]. The CAGR was calculated using data from 2017 to 2019 to forecast antibacterial consumption for the years 2020 to 2022. The 6-year trend analyses were performed on both the actual consumption and forecasted consumption using linear regressions. p values < 0.05 (in bold) were considered as a significant trend. Figures in this table have been rounded for presentation. However, all statistical analyses and significance testing were conducted using unrounded data.

### Sensitivity analysis between ESAC-Net and BeH-SAC data

Absolute values of antibacterial consumption differed between the ESAC-Net dataset (which includes all Belgian hospitals) and the BeH-SAC dataset (limited to acute care hospitals). However, the declining trend in antibacterial consumption (J01) using DID remained significant when considering only the DDD consumed in the acute care hospitals included in BeH-SAC (p = 0.02). For a detailed comparison of the DID values across different hospital groups in the ESAC-Net and BeH-SAC datasets, we refer to Supplementary Table S1. Notably, the decrease in J01 consumption between 2019 and 2022 appeared smaller when considering only acute care hospitals in BeH-SAC (−7.9%) rather than all hospitals in ESAC-Net (−10.9%). We make additional detail on the early and late impact of COVID-19 on antibacterial consumption in DID across different hospital groups available in Supplementary Table S2.

## Discussion

Our main findings indicate that COVID-19 impacted total hospital antibacterial consumption (J01) in Belgium. Uncertainties in treatment, including whether or not COVID-19 patients would profit from receiving antimicrobials to prevent co-infections and from their hypothesised antiviral or anti-inflammatory effects (e.g. azithromycin, an antibacterial agent), prevention policies reducing the prevalence of other infectious diseases, and changes in the demographics of patients during the early pandemic led to a decline in DID, while hospital-based metrics showed an increase. During the pandemic and continuing into 2022, antibacterial consumption measured in DID remained below predicted levels across all subclasses throughout the study period. However, hospital-based metrics approached predicted values, with higher-than-expected consumption for quinolones (J01M), other antibacterials (J01X), tetracyclines (J01A) and penicillin (J01C) using DDD per 1,000 PD, and lower-than-predicted levels for amphenicols (J01B), other β-lactam antibacterials (J01D) and macrolides (J01F).

Between 2020 and 2022, broad-spectrum antibacterial consumption deviated from predicted trends using hospital-based metrics. Piperacillin and enzyme inhibitor (J01CR05), fluoroquinolones (J01MA) and polymyxins (J01XA) exceeded predictions, while third-generation cephalosporins (J01DD) and monobactams (J01DF) dropped below forecasted values.

The impact of the COVID-19 pandemic on hospital antibacterial consumption has been studied across Europe, but it is important to consider that hospital antibacterial consumption is influenced by local policies, such as admission and discharge practices. These differences make direct comparisons between countries challenging. Nevertheless, similar to Hungary, Croatia and Spain, we observed increased consumption using hospital-based metrics between 2019 and 2020 [9,21,22]. Conversely, DID showed decreases similar to Switzerland, Croatia, Iceland, Ireland, Norway and Romania, although this contrasts with countries such as Hungary, Portugal or Italy, where DID increased [9,20,21,23].

Only a few studies have covered AMC data for 2022 thus far. Padullés et al. assessed trends in hospital AMC using DDD per 100 PD between 2008 and 2022 and observed an increasing trend from 2008 to 2010, followed by stable consumption from 2013 to 2017 and from 2018 to 2022 [24]. The latter corresponds to our recent findings. In addition, they observed an increasing trend of piperacillin/tazobactam use based on DDD per 100 PD and a decreasing trend in the use of quinolones, similar to our findings [24]. However, that study reported the largest increase in carbapenem use, whereas in Belgium, consumption declined in 2022 after initial increases from 2019 to 2021.

In parallel with our findings in hospitals, antibacterial consumption in the community decreased with 22% in Belgium [20]. The initial spike in azithromycin consumption in 2020, widely reported across Europe, was driven by its initial, though later unsubstantiated use against COVID-19 [9,21,22]. By 2022, as evidence refuted its efficacy against COVID-19, its consumption had returned to pre-pandemic levels [6].

The disruptive effect of pandemics on antimicrobial prescribing practices has been documented previously by Cortoos et al., who observed deteriorated adherance to guidelines for community-acquired pneumonia therapy during the peak of the influenza A(H1N1)pdm09 pandemic, despite targeted interventions to improve compliance. This highlights the importance of staying vigilant and reinforcing guideline adherence during pandemics [25].

The combined use of DID and hospital-based metrics provides a deeper and more nuanced understanding of antimicrobial consumption trends than using either metric alone. While DDD per 1,000 admissions reflects doses per hospital stay, DDD per 1,000 patient days shows daily antimicrobial exposure. The latter is influenced by hospital discharge policies, such as shifts towards day hospitalisations, increased transmural care and shorter LOS, which can lead to a relative increase in the proportion of hospital days with antimicrobial use. The decrease in LOS might therefore explain the increase in antibacterial consumption between 2019 and 2022 using DDD per 1,000 PD, despite a decrease using DDD per 1,000 admissions.

During the early pandemic, patient days and admissions declined, while Belgium’s population steadily increased, partly explaining the decrease in hospital antibacterial consumption using DID. The reduction in hospitalisations not related to COVID-19, such as elective surgeries often involving prophylactic antibiotics, probably contributed to this decrease. However, the proportion of infection-related hospitalisations increased, resulting in higher antibacterial consumption per hospital stay and a higher proportion of hospitalised patients receiving antibacterials in 2020. From 2021 onward, systemic antibacterial consumption stabilised when measured as DDD per 1,000 patient days, possibly reflecting shorter hospital stays. As expressing consumption in DDD per 1,000 admissions has the advantage of being less affected by shorter hospital stays, the fall in consumption below predicted values in 2022 further indicates fewer antibacterial doses per hospital stay [3].

Rubinić et al. ranked Belgium as the 11th lowest consumer of antibacterials among 24 countries when using DID [11]. With hospital-based metrics, Belgium ranked fifth (DDD/100 bed-days) and sixth (DDD/100 discharges). Austria, the lowest consumer in DDD per 100 bed-days, had an average LOS of 6.2 days in 2021, similar to Belgium [26].

Regarding broad-spectrum antibacterials, consumption of fluoroquinolones (J01MA) declined between 2017 and 2022 using all three metrics, stabilising after 2020 when measured in DID and DDD per 1,000 PD but continuing to decrease using DDD per 1,000 admissions, indicating fewer doses per hospital stay. Consumption of third-generation cephalosporins (J01DD) decreased below predicted levels in 2022 across all three metrics, possibly due to resistance concerns. The ECDC reported that third-generation cephalosporin-resistant *Escherichia coli* and *Klebsiella pneumoniae* caused most disability-adjusted life years (DALYs) and attributable deaths in Belgium in 2020, which probably influenced prescribing practices [27]. Increased use of piperacillin with enzyme inhibitors (J01CR05) may reflect its frequent use for ventilator-associated pneumonia in critically ill COVID-19 patients and the high number of hospital admissions from long-term care facility patients, who are considered at higher risk for resistant bacteria. Both factors probably contributed to offsetting the predicted decline in broad-spectrum antibacterial consumption.

Most observed trends deviated from forecasts based on 2017 to 2019 data. This was likely due to the spike in antibacterial consumption at the beginning of the pandemic, which disrupted the previously observed consumption patterns that had informed the projections using the compound annual growth rate (CAGR). For low consumption subclasses such as amphenicols and polymyxins, relative changes should be interpreted cautiously because their DDD values were low.

This study provides a detailed analysis of antibacterial consumption trends in Belgian hospitals at ATC-2 and ATC-3 levels, as well as for azithromycin, spanning the periods before, during, and in the late phase of the COVID-19 pandemic. By comparing observed vs predicted consumption, it highlights the pandemic's impact and potential lasting changes. Using both population- and hospital-based metrics helps to contextualise trends by accounting for evolving hospital factors.

However, reliance on reimbursement data introduces several limitations. Firstly, there is a 2-year delay in data availability reducing its utility for timely interventions and stewardship efforts. Secondly, non-reimbursed antibacterials are excluded, potentially underestimating total consumption – especially for fluoroquinolones (subject to stricter reimbursement criteria since 2018) and newer broad-spectrum agents such as daptomycin and cefiderocol, not yet approved on the Belgian market [28-30]. Since sales data have already been compared with reimbursement data in the community sector in Belgium, the expected gap is minimal [30]. Thirdly, the data lack crucial details on treatment indications, duration, prescribers, and patient demographics, limiting the ability to evaluate targeted stewardship interventions. Fourthly, reimbursement data include all products dispensed by the hospital pharmacy. In Belgium, hospital pharmacies are not allowed to interfere with community pharmacies, although there is no uniform policy across hospitals. Patients are often provided with medications for home use at discharge, but the proportion of antibiotics dispensed for this purpose is unknown. While no regulations have changed between 2017 and 2022, and we do not expect any meaningful impact, this remains a limitation.

Furthermore, we acknowledge that the ESAC-Net and BeH-SAC datasets cover different hospital groups (n = 170 vs n = 103), with some overlap between them. However, the declining trend of all antibacterials for systemic use (J01) using DID remained significant in a sensitivity analysis.

Finally, shifts in clinical context and patient demographics across COVID-19 periods necessitate cautious interpretation of our findings. In addition, isolating the pandemic’s impact is challenging due to concurrent influences, including ongoing stewardship programmes and reimbursement policies. We also did not include resistance patterns in this study. However, simultaneous surveillance of resistance and consumption trends could enable causal inference.

## Conclusion

A set of population- and hospital-based metrics is essential to provide complementary insights into the complexity of hospital antimicrobial consumption. More granularity is needed for interpretation through additional data on indications, days of therapy or number of different antibacterials used per hospital stay. Patterns of antimicrobial consumption can be profoundly disrupted during unexpected situations, such as pandemics. Unfortunately, the 2-year delay in reimbursement data in Belgium hampers timely feedback to hospitals. Implementing active surveillance systems with timely feedback would enable faster responses, especially during crisis situations.

This study highlights the importance of an appropriate set of denominators to assess trends in hospital antibacterial consumption more accurately. Substantial shifts in consumption patterns during the pandemic underscore the need for continuous monitoring and tailored interventions to optimise antibacterial use in hospitals.

## Data Availability

The data will be made available by the corresponding author upon reasonable request. Access may be subject to institutional or ethical approvals, depending on the nature of the request.

## Supplementary Data

105000 Supplement
