# Supplementary material for: Trends in hospital antibacterial consumption: a retrospective analysis of reimbursement data, Belgium 2017 to 2022
Source: Euro Surveill. 2025 Sep 4;30(35):2500088. doi: 10.2807/1560-7917.ES.2025.30.35.2500088 (PMC12413603; doi:10.2807/1560-7917.ES.2025.30.35.2500088)
Supplement: Supplement [file 25-00088_BONACINI_Supplement.pdf]

Supplementary material

This supplementary material is hosted by *Eurosurveillance* as supporting information alongside the article **Trends in Hospital Antibacterial Consumption in Belgium (2017-2022): a retrospective analysis of reimbursement data**, on behalf of the authors, who remain responsible for the accuracy and appropriateness of the content. The same standards for ethics, copyright, attributions and permissions as for the article apply. Supplements are not edited by *Eurosurveillance* and the journal is not responsible for the maintenance of any links or email addresses provided therein.

Supplementary Table S1 : Comparison of DID in different hospital groups in the ESAC-Net and BeH-SAC datasets.

Year	DID (BeH-SAC)	DID (ESAC-Net)	Absolute Difference	Relative Difference (%)
2017	1.395	1.625	0.23	16.5
2018	1.490	1.628	0.14	9.3
2019	1.351	1.601	0.25	18.5
2020	1.295	1.411	0.12	9.0
2021	1.213	1.435	0.22	18.3
2022	1.245	1.427	0.18	14.6

Supplementary Table S2 : Comparison between different hospital groups (BeH-SAC and ESAC-Net) of early and late impact of COVID-19 on antibacterial consumption in DID.

Relative Change (%)	BeH-SAC (Acute Care Hospitals)	ESAC-Net (All Hospitals)
2019-2020	-4.2	-11.9
2019-2022	-7.9	-10.9
